# Prognostic role of neutrophil-to-lymphocyte ratio to laryngeal squamous cell carcinoma: a meta-analysis^☆^

**DOI:** 10.1016/j.bjorl.2020.09.015

**Published:** 2020-11-09

**Authors:** Yahui Zhao, Jiangbo Qin, Zhaofeng Qiu, Jianzhou Guo, Wei Chang

**Affiliations:** Changzhi Medical College Affiliated Peace Hospital, Changzhi, China

**Keywords:** Laryngeal cell carcinoma, Neutrophil-to-lymphocyte ratio, Overall survival, Disease-free survival, Meta-analysis

## Abstract

**Introduction:**

New evidence suggests that the ratio of neutrophils to lymphocytes is associated with the prognosis of other carcinoma, but the ratio of neutrophils to lymphocytes in laryngeal squamous cell carcinoma remains controversial.

**Objective:**

The objective of this meta-analysis was to clarify the prognostic effectiveness of the ratio of neutrophils to lymphocytes in laryngeal squamous cell carcinoma.

**Methods:**

According to the meta-analysis of the free guide, we searched EMBASE, Pubmed, the Cochrane Library databases. The ratio of neutrophils to lymphocytes of laryngeal squamous cell carcinoma patients was evaluated using mean standard vehicle and confidence interval. The overall survival, disease-free survival and progression free survival of patients with laryngeal squamous cell carcinoma were expressed by standard mean carrier method and confidence interval. The risk ratio of 95% confidence interval was used as an evaluation index for patients with laryngeal squamous cell carcinoma.

**Results:**

Eight studies, including 1780 patients, used a variety of different end values to classify the ratio of neutrophils to lymphocytes (range 1.78–4.0). Among the eight studies that reported risk ratio of the overall survival, the higher median value was 2.72, and 2 of 4 studies reported disease-free survival results. The critical value of ratio of neutrophils to lymphocytes and overall survival deterioration (risk ratio = 1.68, 95% confidence interval 1.43–1.99, *p* < 0.001), disease-free survival (risk ratio = 2.09, 95% confidence interval 1.62–2.6, *p* < 0.001) and progression free survival (risk ratio = 1.92, 95% confidence interval 1.75–2.10, *p* < 0.001) was associated with with laryngeal aquamous cell carcinoma. The ratio of neutrophils to lymphocytes had prognostic value for laryngeal squamous cell carcinoma.

**Conclusion:**

The results of this meta-analysis showed that the increase of neutrophils to lymphocytes ratio was related to poor prognosis of laryngeal squamous cell carcinoma. The neutrophils to lymphocytes ratio may serve as a cost-effective prognostic biomarker of poor prognosis of laryngeal squamous cell carcinoma. More high-quality prospective trials are needed to assess the practicability of evaluating the ratio of neutrophils to lymphocytes in laryngeal squamous cell carcinoma.

## Introduction

Laryngeal cell carcinoma is the second most prevalent malignant tumor after non-cervical cancers. About 1,700,000 cases of laryngeal cell carcinoma are reported every year in the world, and near 90,000 die due to this condition. Differences between ethnic groups, countries, ages and genders are responsible for different rates of morbidity.^1^ The pathological mechanism of LSCC is not clear; factors such as smoking, drinking, air pollution, and HPV are known to be inductors of this disease. The treatment of laryngeal cell carcinoma can be very disappointing due to the differences in clinical staging, behavior and prognosis. While multimodal therapy, including surgery, radiotherapy, chemotherapy, has improved, it is still disappointing because of its anatomical structure specificity and lack of prognosis.^2^ It is necessary to identify biomarkers for overall survival (OS), progression survival (PFS), and disease-free survival (DFS) in patients with laryngeal squamous cell carcinoma. In clinical practice, the short-term and long-term prognosis of laryngeal squamous cell carcinoma depends on its clinical stage, distant metastasis, primary tumor size and lymphatic involvement. Because different stages of the disease have their own characteristic, the staging fact alone are not enough to predict the prognosis and recurrence risk of laryngeal squamous cell carcinoma. In recent years, the prediction of laryngeal squamous cell carcinoma by laboratory blood indexes has been the focus of research, involving occurrence, development, invasion and metastasis of several types of cancer.^3^ Some laboratory inflammatory markers have been identified as prognostic markers for specific cancers, such as neutrophil granulosa cells, T lymphocytes, B lymphocytes, prominent cells, natural killer cells and mast cells, etc.^4^ These inflammatory markers can produce chemokines, inflammatory mediators and cytokines, and their tumor–host interactions lead to systemic inflammatory responses. Recently, high NLR values have been proved to be an important prognostic factor in colorectal cancer, breast cancer, renal cell carcinoma and ovarian cancer.^5^ But the relationship of prognosis of laryngeal squamous cell carcinoma and an elevated NLR remains controversial. Bojaxhiu has studied NLR levels in 186 patients with laryngeal squamous cell carcinoma and found that elevated NLR level (NLR greater than 3.28) was associated with overall survival (OS), but no correlation was found with local recurrence-free survival (RFS).^6^ Chen believes that preoperative NLR (NLR > 2.45) is significantly associated with survival rate and tumor progression in patients with laryngeal squamous cell carcinoma, which may be an independent prognostic indicator for postoperative OS and PFS of laryngeal squamous cell carcinoma.^7^ Also, Song studies, showed that NLR (>1.85) had a significant correlation with PFS, but no correlation with OS.^8^ Because distinct researches have used different optimal critical values of NLR, this fact may be a factor that affects the interpretation of the results. Therefore, an elevated NLR as a predictor of recurrence and prognostic index of laryngeal squamous cell carcinoma is still incipient. The purpose of this meta-analysis is to clarify the influence of NLR in the prognosis of this clinical condition.

## Methods

### Literature search strategy

Articles reported in the literature with NLR as prognostic and recurrence factors of laryngeal squamous cell carcinoma were included in this study. To avoid the omission of original materials, we used a database and keywords for a comprehensive search. The database consists of EMBASE, Pumbed and the Cochrane Library, keywords including “laryngeal carcinoma”, “Neoplasms Laryngeal”, “Laryngeal Neoplasm”, “Lar. ynx Neoplasms”, “Neoplasm Larynx”, “Laryngeal Cancer”, “Neutrophil-to-Lymphocyte Ratio”, “neutrophil to lymphocyte ratio”, “neutrophils”, “lymphocytes”, “NLR”. Operated by AND or OR connection, the retrieval deadline is set as May 2020. At the same time, we also explored the references to avoid missing the relevant literature. We first searched the article title and abstract to exclude the documentation that was not relevant or repeated, and screened the full read-through which included laryngeal squamous cell carcinoma and NLR.

### Literature inclusion and exclusion criteria

Two authors (ZFQ and WC) reviewed and retrieved the original study in all the literature, and the disagreement was discussed or resolved by third-party researchers (JBQ). We developed detailed inclusion and exclusion criteria. Inclusion criteria: (1) all studies should contain a relationship between preoperative peripheral blood NLR levels and prognosis in laryngeal squamous cell carcinoma. (2) Interventions such as surgery, radiotherapy, chemotherapy, or combination therapy should have relevant survival data and prognosis prediction including risk ratio (HR) and 95%CI or OS, PFS, DFS (3) subjects were all humans, and all articles were written in English language. (4) All laryngeal squamous cell carcinoma participants should have been excluded from possible predisposing factors (e.g., tumor, esophageal cancer, etc.) or of taking drugs that could be associated with development of laryngeal squamous cell carcinoma. Exclusion criteria: (1) duplicate literature, case reports, not written in English, abstracts, conference papers found by the database; (2) if the study treatment plan of the article was distinct from the above treatment plan; (3) other diseases than the subject of the study, laryngeal squamous cell carcinoma.

### Data extraction

The extracted information included the NLR level values of the included studies, year of publication, sample size, age (mean ± standard deviation), smoking, alcohol consumption, follow-up, and OS, RFS, DFS of laryngeal squamous cell carcinoma.

The systematic review was initially carried out by two reviewers (ZFQ and WC) in accordance with the newcastle-ottawa (NOS).^9^ The highest score was 9 and score >5 was considered as high quality. Cases of ambiguity, were evaluated by a third party (JBQ). We used the Review Manager 5.3 for Meta- analysis. Effect model was selected according to the P-value of the chi-square test. Literature heterogeneity size assessment was measured based on I^2^ values. I^2^ range in 75%–100% was considered high; I^2^ range in 50%–75% considered medium; I^2^ range in 25%–50% considered as low heterogeneity; and percentages lower than that were considered as non-heterogeneity. I^2^ > 50% is a fixed-effect model, and I^2^ < 50% is a random effect model. Sensitivity analysis was achieved by removing each of the individual items, and its weight in the total effect.

## Results

**Figure 1:**
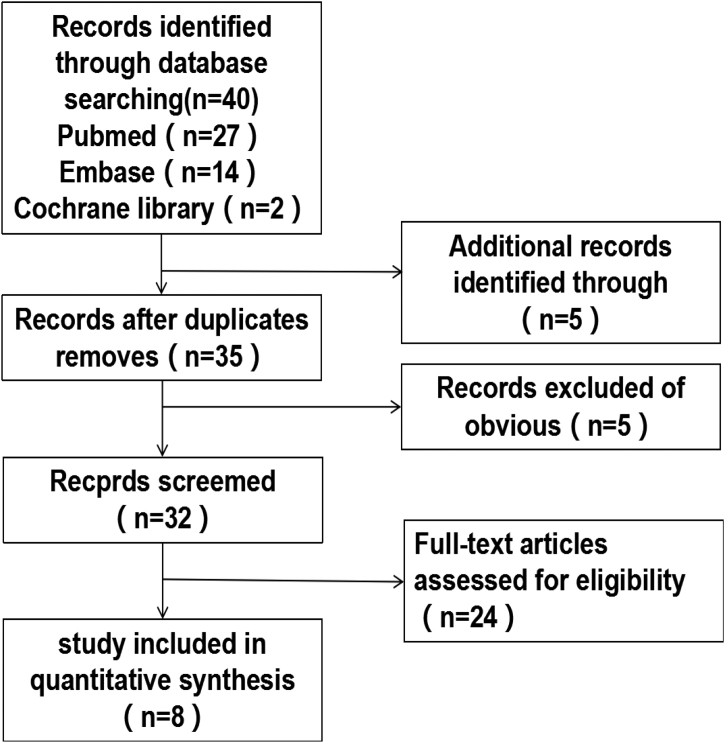
Literature screening flowchart.

After including studies according to quality assessment we retrieved 40 original studies exploring strategies using electronic databases. First, we excluded five duplicate documents or articles written by the same scholar or institution. All the remaining articles were selected on the basis of the pre-stablished exclusion and inclusion criteria, and we finally had eight included articles for this research,^7^, ^10^, ^11^, ^12^, ^13^, ^14^, ^15^, ^16^ the retrieval process is shown in Fig. 1, and the detailed basic characteristics of the articles are shown in Table 1.

**Table 1:** Characteristics and quality assessment results for each included publication.

Study	Time	Country	Ethnicity	Patients (male/female)	Age (range)	Result	Follow-up (month)	Primary location			Cutoff	NOS score
Chen et al.^7^	2018	China	Asia	361 (353/8)	60 (35−87)	OS PFS	47 (4−98)	280 (77.56%)	70 (19.39%)	11 (3.05%)	2.45	7
Song et al.^8^	2019	China	Asia	137 (133/4)	62 (40–84)	OS RFS	47 (2–111)	Glottic: 61 (44.5%) supraglottic and subglottic: 76 (55.4%)			2.96	8
Tu et al.^10^	2015	China	Asia	141 (137/4)	59 (36–87)	OS DFS	51 (5−102)	24 (17%)	113 (80.2%)	4 (2.8%)	2.17	8
Zhou et al.^11^	2019	China	Asia	232 (192/40)	63 (39−81)	OS DFS	27.3 ± 18.6	68 (29.3%)	152 (65.2%)	12 (5.2%)	2.38	7
Fu et al.^12^	2016	China	Asia	420 (413/7)	60 ± 9.1 (33−84)	OS CSS		198 (47.14%)	206 (49.04%)	16 (3.82%)	2.59	8
Wong et al.^13^	2016	United Kingdom	Europe	140 (121/19)	66 (36−92)	OS DFS	41.5 (2−103)				＜1.78 2.41 3.10 ＞3.10	5
Wang et al.^14^	2016	China	Asia	120 (118/2)	60.6 ± 8.6 (40−81)	OS PFS	40.1 ± 14.9	52 (43.33%)	63 (52.50%)	5 (4.17%)	2.79	6
Aires et al.^15^	2018	Turkey	Asia	229 (221/8)	59 (31−88)	OS DFS PFS	39.5 (1–107)	79 (40.97%)	82 (50.93%)		4	6

### Meta-analysis results

**Figure 2:**
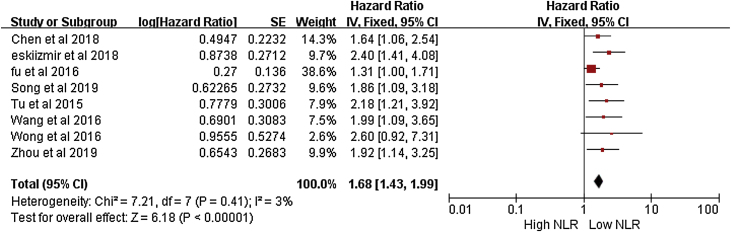
Overall survival rate and NLR ratio in LSCC.

A meta-analysis of overall survival rate and NLR ratio in patients with LSCC was performed. The forest plot results of 8 studies on the rate of overall survival to NLR is shown in Fig. 2.^7^, ^10^, ^11^, ^12^, ^13^, ^14^, ^15^, ^16^ The meta-analysis included the overall survival (OS) rate of LSCC and its correlation with NLR. The HR was 1.68 (95% CI = 1.43–1.99), which was statistically significant. Due to the low heterogeneity of the study (I^2^ = 5), the fixed effect model wad used in Fig. 2.

### Meta-analysis of RFS and NLR ratio in patients with LSCC

**Figure 3:**
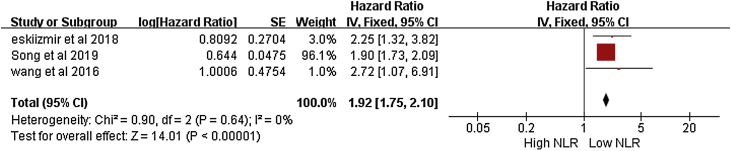
DFS and NLR ratio in LSCC.

The results show three studies with rate of RFS related to NLR in laryngeal squamous cell carcinoma.^13^, ^14^, ^16^ The meta-analysis showed that the RFS of laryngeal squamous cell carcinoma was related to NLR. Namely HR = 1.92 (95% CI 1.75–2.10; *p* < 0.001), (Fig. 3), with statistical significance, a fixed-effect model was used because of the low heterogeneity of the study (I^2^ = 0).

### Meta-analysis of DFS and NLR ratio in patients with LSCC

**Figure 4:**
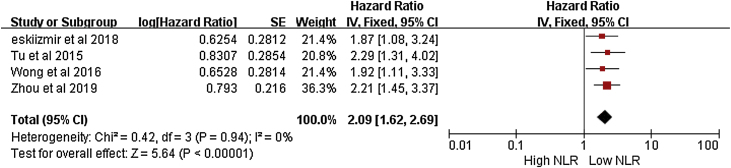
RFS and NLR ratio in LSCC.

The results of four studies on the rate of DFS related to NLR in LSCC are shown in Fig. 4.^10^, ^11^, ^13^, ^16^ The meta-analysis showed that the DFS of laryngeal squamous cell carcinoma was related to the values of NLR. Namely HR = 2.09 (95% CI 1.62–2.69; *p* < 0.001), with statistical significance, a fixed effect model was used because of the low heterogeneity of the study (I^2^ = 0) in Fig. 4.

### Quality evaluation

The results of the assessment of literature quality level are summarized in Table 1. The statistical quality level in three was high, four were medium and one, low.

## Discussion

To demonstrate the prognostic value of preoperative elevated NLR with laryngeal squamous cell carcinoma, we performed a meta-analysis by searching and screening the published literature. A total of 8 studies and 1780 patients were included and the clinical importance of elevated value of preoperative NLR in the prognosis of laryngeal squamous cell carcinoma was evaluated. Our study showed that preoperative high value of NLR index was associated with the OS, DFS and PFS of laryngeal squamous cell carcinoma, NLR was a good prognostic marker of laryngeal squamous cell carcinoma.

A number of literature studies have shown that NLR have been confirmed to have prognostic predictive effects on other cancers, such as breast cancer, renal cell carcinoma, colorectal cancer, myeloma, etc. The relationship between OS, DFS, RFS and NLR of laryngeal squamous cell carcinoma in our study is also consistent.

The prognostic mechanism of preoperative elevated NLR on OS, DFS, RFS of laryngeal squamous cell carcinoma is still unclear. One possible mechanism among the general immune response is the involvement of neutrophils. Neutrophils have different effects in different stages of tumor.^17^ They are an important line of defense to prevent infection and anti-inflammatory, but can also promote tumor invasion and metastasis. This may be associated with the presence of anti-inflammatory (N1) and pro-inflammatory (N2) phenotypes in neutrophils. The N2 type has a high level of arginase 1 (arginase1, ARG1), which can induce the production of N0 enzymes, which inhibit the cell activity CD8T antitumor and promote the development of tumor;^18^ Another mechanism is related to the T lymphocytes, which have more types and functions and play a major role in inflammatory response. It is divided into four types: killing, inhibiting, inducing auxiliary and delayed hypersensitivity.^19^ Different types of tumors have specific antigens and can be recognized and killed by T lymphocytes.^20^ Malignant tumor-related inflammation inhibits antitumor immunity and promotes tumor growth and metastasis by mobilizing regulatory T cells and activating chemokines. Hence, NLR may be considered as a potential clinical indicator of lymphocytes and neutrophils.^21^ Moreover, it is a low-cost and readily available and effective indicator in the clinical set, thus making it the most potential prognostic marker for laryngeal squamous cell carcinoma.^22^

In addition, 6 of the 8 articles in our study were Chinese, and there were no regional grouping analysis, so, they might have had a selection bias; most cases of laryngeal squamous cell carcinoma were male patients, mainly determined by the characteristics of the disease itself, they may have also had a gender selection bias; the cut-off level of NLR would need to be verified by various methods in the included studies. The influence of many factors, such as country, region, institution, and different cut-off values, makes the reference standard inconsistent. Due to the different cut-off values, making difficult to know what would be the best cut-off range and how to interpret the results, we set the range between 1.78 and 4. There were 7 study items between 2 and 3, and only one item reached 4. Inconsistent cut-off values may have a selection bias. In future researches, we need to set a unified scope to facilitate the big data statistical analysis. Some recent studies have shown that other inflammatory factors, such as C-reactive proteins, may have a preclinical significance of laryngeal squamous cell carcinoma. Whether NLR interact with other inflammatory factors is unclear and needs further verification by the big data. Furthermore, the correlation between NLR and some clinical diseases, such as coronary heart disease, obstructive sleep apnea hypopnea syndrome, also needs further clinical verification.

Despite the above limitations, our meta-analysis still has some advantages. First, our data were analyzed by a single factor and multivariate analysis, and a preoperative elevated value of was related to a good prognostic significance in laryngeal squamous cell carcinoma patients and avoided the influence of other inflammatory factors. Besides, NLR is a laboratory index, which is costless, easy to obtain and very common in the clinical set. In addition, our study determined a good predictor of laryngeal squamous cell carcinoma. Because we provided relatively few prospective studies, more clinical big data information is needed to further demonstrate NLR prognostic significance in screening high-risk patients with laryngeal squamous cell carcinoma.

## Conclusion

The results of meta-analysis showed that the increase of NLR was related to poor prognosis of LSCC. NLR may serve as a cost-effective prognostic biomarker of the poor prognosis of LSCC. More high-quality prospective trials are needed to assess the practicability of NLR in LSCC.

## Conflicts of interest

The authors declare no conflicts of interest.
